# Staphyloraphy

**Published:** 1870-01

**Authors:** 


### ARTICLE IX.

### *Staphyloraphy.*

In a report on this subject to the Illinois State Medical Society, Dr. Moses Gunn, of Chicago, gives some interesting statistics. He says:

Prof. Mussey, of Cincinnati, reports four operations, all on the soft palate. In one case, the patient attained perfect articulation; in another there was improvement; two were lost sight of.

Prof. Goldsmith, now of Vermont, reports seven operations, six of which were upon the velum, and one upon the entire palate; there was improvement in speech in five.

Prof. Hodgen, of St. Louis, reports three operations; one on complete fissure, and two on fissure of velum only. In one of the latter two there was decided improvement. In the first case mentioned, an obturator was being fitted at the time of writing.

Prof. Miner, of Buffalo, reports three operations, two for incomplete, and one for complete, fissure. In the latter, an obturator was used. Result—decided improvement in one case.

Prof. Marker, of New York, reports two operations, one of which was for congenital, and one for traumatic, fissure. In the congenital case, there was very slight improvement, while in the traumatic case, there was a perfect restoration of speech. These two cases are especially interesting and instructive, illustrating, as they do, the difference between the recovery of a temporarily lost power and the acquiring of an entirely new art, after maturity, and, at best, with but an imperfect organ.

Prof. Gross writes:—"I do not think that the speech ever improves very greatly after the operation, however successful. This, certainly, has been the result of my own experience, notwithstanding the pains which most of my patients have taken to educate themselves in articulation."

Prof. Parker has operated four times, in all instances, for fissure of velum only. He writes:—"In each case I was very much disappointed in the result upon articulation. There was no decided improvement. My disapointment was so great as regards improvement in speech that for many years I have refused to operate."

Your reporter can from his own experience enumerate only three successful operations, and honesty compels him to acknowledge that those were of no benefit to speech.

A little reflection on the subject will enable us to see why so small rewards should attend, or rather follow upon, this operation. The operation requires for its performance the cooperation of the patient; this is inconsistent with either an anæsthetic condition or that lack of courage and endurance which, as a general rule, characterizes childhood. Consequently, we are compelled to postpone surgical interference till about the period of dawning maturity. And, now, after a successful operation, at this late period, the poor unfortunate attempts to learn a new and really difficult art, and that, too, with an imperfect apparatus—a machine imperfect in one of its important constituents. The difficulties which attend his efforts, and which he must overcome, may be faintly appreciated when, after maturity, we attempt to articulate a new language, or correct, here and there, in our native tongue, a long-practised habit of incorrect pronunciation. The German, however intellectual by nature or cultivated by study he may be, finds it almost impossible to articulate some of the sounds of our language, as, for instance, the sound of *th*, while the American finds it equally difficult to express correctly the gutterals of the German. Add to these well-known difficulties a habit grown and matured with the individual of misarticulating each and every articulate element of a language; consider, also, that his machinery for articulation, though materially improved by the operation, is still far from perfect; remember how small is the proportion of really tractable or persevering men, and we shall cease to wonder at the small number who, after this operation, ever attain fair powers of articulation.

As a merely surgical procedure, staphyloraphy is a feasable operation; but as to its rewards, a reasonable doubt may yet be entertained.—*Med. & Surg. Reporter.*

---

